# Analysis of the Dose-Response Effects of Physical Activity on Cardiocerebrovascular and All-Cause Mortality in Hypertension

**DOI:** 10.3389/fcvm.2022.844680

**Published:** 2022-03-17

**Authors:** Jun-Peng Xu, Rui-Xiang Zeng, Hai-Ning Lu, Yu-Zhuo Zhang, Xiao-Yi Mai, Shuai Mao, Min-Zhou Zhang

**Affiliations:** ^1^The Second Clinical College, Guangzhou University of Chinese Medicine, Guangzhou, China; ^2^Department of Critical Care Medicine, Guangdong Provincial Hospital of Chinese Medicine, Guangzhou, China

**Keywords:** physical activity, mortality, hypertension, dose-response relationship, cardiocerebrovascular

## Abstract

**Background:**

Leisure-time moderate-to-vigorous physical activity (MV-PA) has been consistently regarded as a protective factor to prevent and treat hypertension. However, the effect of different levels of MV-PA against cardiocerebrovascular and all-cause mortality in hypertension is still unclear. The aim of this study was to explore the dose relationships of MV-PA on these adverse outcomes in hypertension.

**Methods:**

In the National Health and Nutritional Examination Survey (NHANES) from 1999 to 2006, participants with hypertension were enrolled and classified into inactive (0 MET-h/week), low-active (0 < to < 7.5 MET-h/week), and high-active (≥ 7.5 MET-h/week) groups. A multivariate Cox regression analysis was conducted with a hazard ratio (HR) and corresponding 95% confidence interval (CI). To further explore the association between different levels of MV-PA and adverse outcomes, Kaplan-Meier survival curves, subgroup analysis, and restricted cubic spline curves were performed.

**Results:**

During a median 10.93-year follow-up, 1,510 and 347 patients had died from any causes and cardiocerebrovascular, respectively. The high-active group had the highest event-free survivals of all outcomes compared with low-active and inactive groups. A multivariate Cox regression analysis demonstrated that the high-active and low-active groups were associated with reduced risks of all-cause [HR: 0.70, 95% CI: 0.60–0.82; 0.76 (0.68–0.86), respectively] and cardiocerebrovascular mortality [0.56 (0.41–0.77); 0.63 (0.50–0.81), respectively] compared with the inactive group. Subgroup analysis and restricted cubic spline curves showed that MV-PA surpassing 15 MET-h/week could decrease the risks of cardiovascular and all-cause mortality with inverse relationships, which was not the case for cerebrovascular mortality, indicating a U-shaped association.

**Conclusion:**

Our study suggests that highly active MV-PA of 7.5 to < 15 MET-h/week was associated with the lowest risks of cardiocerebrovascular and all-cause mortality in hypertension.

## Introduction

An estimated 1.13 billion people are living with hypertension worldwide (1). The current updated report indicates that total direct and indirect costs from 2010 to 2030 attributed to hypertension are projected to be US$400 billion (2). Although the treatment measures of hypertension have been improved in recent decades, there is much room for further improvement, as hypertension is not accompanied by obvious relevant symptoms and, like a silent killer, causes various cardiocerebrovascular complications, or even death (1). Hypertension manifests a chronic state of increased autoimmunity, inflammation, and oxidative stress, which eventually leads to vascular dysfunction (3). Conversely, regular physical activity (PA) confers several cardiocerebrovascular benefits on the improving mitochondrial antioxidant system and lipid metabolism and reducing systemic inflammation and endothelial dysfunction (4, 5).

Substantial evidence supports that moderate-to-vigorous PA (MV-PA) can prevent and treat hypertension (6, 7) or even lower risks of adverse cardiocerebrovascular events and mortality in the general population (8). The latest 2020 European Society of Cardiology (ESC) Guidelines on Sports Cardiology and Exercise in Patients with Cardiovascular Disease also underline that regular exercise plays an important role in therapy for hypertension and recommend at least 30 min, 5–7 days per week of moderate PA [(corresponding to a minimum of 7.5 metabolic equivalent hours per week, (MET-h/week)] (9). Additionally, two large-scale cohort studies of the UK Biobank and Korean National Health Insurance Service showed similar results: 1–7 days per week of MV-PA was beneficial in reducing major adverse cardiocerebrovascular events and all-cause death among patients with and without controlled hypertension compared with those without any MV-PA (8). Nonetheless, they failed to determine prominent dose-response relationships between the frequencies of MV-PA and the prespecified outcomes. In short, in the hypertensive population, previous evidence offered support for the maintenance of a modest amount of MV-PA in strategic planning for adverse cardiocerebrovascular events and all-cause death prevention and intervention, but the detailed dose-response relationships between MV-PA and adverse outcomes are not yet known.

To further assess the prognostic effect of MV-PA on cardiocerebrovascular and all-cause mortality in hypertension, this study explored their dose-response relationships in a large longitudinal cohort with a hypertensive population.

## Materials and Methods

### Study Population

The National Health and Nutritional Examination Survey (NHANES) is a series of national surveys to evaluate the health status of US citizens with a complex, stratified, multistage, and probability sampling method. The Centres for Disease Control and Prevention ratified the study protocols, and all participants provided informed consent. Details about the surveys and corresponding death index are available at www.cdc.gov/nchs/nhanes and www.cdc.gov/nchs/ndi/, respectively. We abstracted the data from 1999 to 2006 of the NHANES into this study.

**FIGURE 1:**
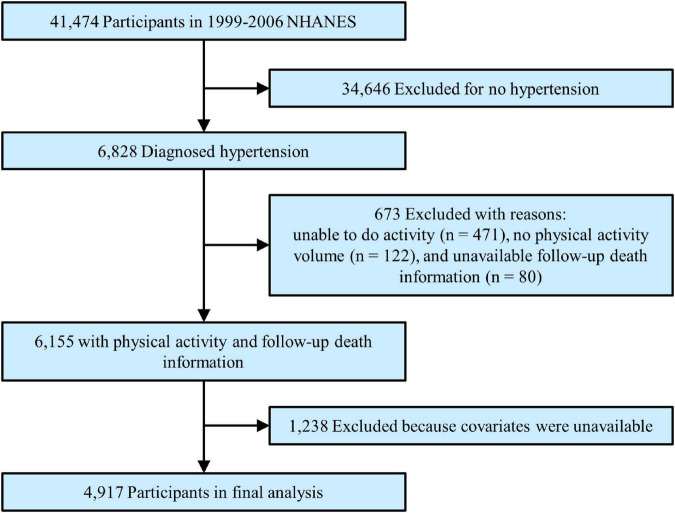
Study flowchart. Flowchart showing the process of participant selection. Of 41,474 participants from 1999 to 2006 National Health and Nutrition Examination Survey (NHANES), 4,917 remained in the final analysis.

The study population was adult patients with hypertension with complete data on leisure-time PA (i.e., moderate and/or vigorous PA), follow-up death index, and covariates. Hypertension was determined by specialist doctors when a subject had a diagnosed hypertension history, or non-same-day randomised records of 3 times systolic blood pressure ≥ 140 mmHg or diastolic blood pressure ≥ 90 mmHg, or was taking antihypertensive drugs. Each blood pressure was calculated as a mean value from three consecutive right-hand readings of a mercury sphygmomanometer after resting quietly in a sitting position for 5 min. The total number of participants in the primary survey was 41,474. After excluding participants without hypertension (*n* = 34,646), who were unable to perform any PA (*n* = 471), who had unavailable MV-PA data (*n* = 122), without follow-up death information (*n* = 80), or without covariate data (*n* = 1,238), a total of 4,917 patients were enrolled in the final analysis (Figure 1).

### Physical Activity Assessment

Detailed information about MV-PA was listed in the NHANES PA questionnaire. The types, frequency, duration, and intensity of leisure-time MV-PA over the past 30 days were recorded. The types of MV-PA included over 61 exercises, such as aerobics, baseball, basketball, bicycling, dance, football, golf, running, swimming, treadmill, yoga, martial arts, and wrestling. In addition, the MV-PA volume of each exercise was listed in PA codes. According to Ainsworth’s Compendium of PA, the intensity of each MV-PA type was converted into a metabolic equivalent of task (MET), which is an objective measurement of the energy expenditure of a specific activity (10). First, the MV-PA volume for each patient was computed by multiplying the intensity (MET), frequency, and duration (h) of the activity over the past 30 days. Second, patients were categorised into inactive (0 MET-h/week), low-active (0 < to < 7.5 MET-h/week), or high-active (≥ 7.5 MET-h/week) groups in response to the European Society of Cardiology and World Health Organisation recommendation of at least 30 min, 5–7 days per week of moderate-intensity PA or an equivalent combination with vigorous-intensity PA (corresponding to 7.5 MET-h/week), for the general hypertensive population (9, 11).

### Variables and Follow-Up Outcomes

The independent variable in this study was MV-PA volume. Dependent variables were follow-up adverse outcomes, such as cardiovascular, cerebrovascular, cardiocerebrovascular, and all-cause mortality. Cardiocerebrovascular mortality was defined as total incidents of cardiovascular and cerebrovascular deaths.

The following covariates were included in the analysis: age, gender, race, body mass index, education, smoking, diabetes, coronary heart disease, chronic heart failure, stroke, cancer, systolic blood pressure, diastolic blood pressure, heart rate, total cholesterol, triglycerides, low-density lipoprotein cholesterol (LDL-C), estimated glomerular filtration rate (eGFR), cholesterol intake, total fat intake, energy intake, protein intake, sodium intake, and medications (such as antiplatelet, warfarin, statin, antihypertensive drugs, β-blockers, α-blockers, diuretics, and hypoglycaemic agents). Except for eGFR, the detailed acquisition process and measuring method of each variable are available at www.cdc.gov/nchs/nhanes. eGFR was estimated using the Chronic Kidney Disease Epidemiology Collaboration equation and was used to evaluate the kidney function of each participant (12). Chronic kidney disease was defined as eGFR < 60 ml/min/1.73 m^2^.

### Statistical Analysis

Baseline characteristics of the included participants were divided by MV-PA volume (0, 0 < to < 7.5, and ≥ 7.5 MET-h/week), with the continuous variables reported as the mean ± SD and the categorical variables reported as numbers with percentages. To detect the differences between groups classified by MV-PA volume, χ^2^ tests or Kruskal–Wallis tests were performed, where appropriate Kaplan-Meier plots were generated to estimate the event-free survival, and log-rank tests were applied to examine the different groups of MV-PA. Cox proportional hazards models were used to analyse the associations between MV-PA and study outcomes with different adjustments. Model I was not adjusted for any confounders, and Model II was adjusted for age, gender, and race. Model III was fully adjusted for body mass index, education, smoking, diabetes, coronary heart disease, chronic heart failure, stroke, cancer, systolic blood pressure, diastolic blood pressure, heart rate, total cholesterol, triglycerides, LDL-C, eGFR, cholesterol intake, total fat intake, energy intake, protein intake, sodium intake, and medications in addition to the adjusted confounders of model II.

Additionally, several subgroup and sensitivity analyses were performed by a stratified multivariate regression analysis. First, we conducted different subgroups, such as age, gender, race, diabetes, coronary heart disease, cancer, chronic kidney disease, and antithrombotic therapy, to identify potential effect modifiers. To test for statistical significance of interactions, interaction terms between different groups of MV-PA and different subgroups were generated and examined by the Wald test for dichotomous variables and the likelihood ratio test for multilevel variables. If necessary, the possible interactions between all adjusted factors were also tested. Second, we used restricted cubic spline curves with knots at 5, 35, 65, and 95 percentiles of MV-PA to explore the predictability and dose-response relationships of MV-PA as a continuous variable. Third, the MV-PA groups were redefined to test the robustness of the results based on different MET-h/week cut-offs. Fourth, to control the imbalance of baseline covariates, we performed a propensity score matching that included all covariates in the fully adjusted Cox models. A value of *p* < 0.05 (two-sided) was considered statistically significant. All analyses were performed with EmpowerStats software^1^ except for the restricted cubic spline curves, which were calculated by the *ggplot2* and *rms* packages for R (version 4.0.3).

## Results

### Baseline Characteristics

Table 1 presents the baseline characteristics of a total of 4,917 hypertensive patients enrolled in the study. Among all the participants, 48.2% were men, over 50% were white, and the mean age at enrolment was 59.53 years old. Overall, there were significant differences in baseline characteristics between the inactive, low-active, and high-active groups, with the exception of body mass index, total cholesterol, LDL-C, taking antiplatelets, and taking α-blockers. Compared to other groups, participants in the high-active group had fewer comorbidities, such as diabetes, coronary heart disease, chronic heart failure, stroke, and cancer, with lower rates of statin use, antihypertensive drugs, warfarin, β-blockers, diuretics, and hypoglycaemic agents. Despite increased cholesterol, total fat, energy, protein, and sodium intake, participants in the high-active group were more likely to take control of serum lipids and blood pressure. During the 10.93-year of the median follow-up period, 1,510 (30.71%), 296 (7.99%), and 51 (3.50%) patients died from any causes, cardiovascular, and cerebrovascular, respectively.

**TABLE 1:** Baseline characteristics of study participants by physical activity^a^ (PA).

Characteristics	All (*n* = 4917)	Physical activity			
		Inactive (*n* = 2260)	Low-active (*n* = 1412)	High-active (*n* = 1245)	*P*-value
Male	2371 (48.22)	998 (44.16)	678 (48.02)	695 (55.82)	<0.001
Age, years	59.53 ± 16.00	61.39 ± 15.48	61.50 ± 15.60	53.93 ± 16.09	<0.001
Body mass index, kg/m^2^	30.30 ± 6.73	30.59 ± 7.13	30.19 ± 6.42	29.88 ± 6.29	0.082
**Race**					<0.001
Black	1184 (24.08)	622 (27.52)	279 (19.76)	283 (22.73)	
White	2580 (52.47)	1014 (44.87)	819 (58.00)	747 (60.00)	
Other	1153 (23.45)	624 (27.61)	314 (22.24)	215 (17.27)	
**Education**					<0.001
Lower than high school	1620 (32.95)	998 (44.16)	422 (29.89)	200 (16.06)	
High school	1231 (25.04)	584 (25.84)	367 (25.99)	280 (22.49)	
More than high school	2066 (42.02)	678 (30.00)	623 (44.12)	765 (61.45)	
**Smoking**					<0.001
Never smoker	2380 (48.40)	1087 (48.10)	688 (48.73)	605 (48.59)	
Current smoker	1644 (33.44)	695 (30.75)	504 (35.69)	445 (35.74)	
Ex-Smoker	893 (18.16)	478 (21.15)	220 (15.58)	195 (15.66)	
Diabetes	1058 (21.52)	548 (24.25)	327 (23.16)	183 (14.70)	<0.001
Coronary heart disease	849 (17.27)	430 (19.03)	277 (19.62)	142 (11.41)	<0.001
Chronic heart failure	286 (5.82)	153 (6.77)	99 (7.01)	34 (2.73)	<0.001
Stroke	321 (6.53)	163 (7.21)	102 (7.22)	56 (4.50)	0.004
Cancer	654 (13.30)	282 (12.48)	230 (16.29)	142 (11.41)	<0.001
Systolic blood pressure, mmHg	128.71 ± 15.03	130.02 ± 16.32	127.79 ± 13.46	127.38 ± 14.06	<0.001
Diastolic blood pressure, mmHg	73.84 ± 12.80	73.69 ± 13.49	72.92 ± 12.28	75.16 ± 11.95	<0.001
Pulse pressure, mmHg	54.87 ± 12.83	56.33 ± 13.53	54.86 ± 12.29	52.22 ± 11.68	<0.001
Heart rate, beats per minute	72.04 ± 12.72	72.63 ± 12.63	71.61 ± 12.29	71.44 ± 13.32	0.016
Total cholesterol, mg/dl	205.02 ± 43.54	206.29 ± 44.38	203.25 ± 42.48	204.70 ± 43.14	0.115
Triglyceride, mg/dl	162.11 ± 86.61	164.24 ± 93.80	162.29 ± 78.68	158.02 ± 81.34	0.023
LDL-C, mg/dl	118.95 ± 24.97	118.95 ± 25.04	118.95 ± 25.30	118.95 ± 24.46	0.939
eGFR, ml/min/1.73 m^2^	84.38 ± 25.45	83.78 ± 26.74	82.04 ± 24.84	88.12 ± 23.24	<0.001
Cholesterol intake, mg	275.15 ± 203.36	270.11 ± 199.44	267.64 ± 193.05	292.80 ± 220.24	0.002
Total fat intake, gm	72.40 ± 39.92	69.10 ± 38.57	71.82 ± 39.24	79.04 ± 42.27	<0.001
Energy intake, kcal	1918.58 ± 854.95	1839.50 ± 840.30	1892.51 ± 825.07	2091.71 ± 889.94	<0.001
Protein intake, gm	74.95 ± 35.91	70.88 ± 35.46	74.32 ± 33.80	83.06 ± 37.68	<0.001
Sodium intake, mg	3009.00 ± 1139.72	2941.76 ± 1157.25	3023.50 ± 1133.09	3114.63 ± 1106.97	<0.001
Antiplatelet	167 (3.40)	81 (3.58)	55 (3.90)	31 (2.49)	0.109
Warfarin	115 (2.34)	64 (2.83)	37 (2.62)	14 (1.12)	0.004
Statin	894 (18.18)	365 (16.15)	301 (21.32)	228 (18.31)	<0.001
Antihypertensive drugs	1643 (33.41)	776 (34.34)	499 (35.34)	368 (29.56)	0.003
β-blockers	401 (8.16)	175 (7.74)	135 (9.56)	91 (7.31)	0.066
α-blockers	146 (2.97)	65 (2.88)	48 (3.40)	33 (2.65)	0.493
Diuretics	940 (19.12)	455 (20.13)	298 (21.10)	187 (15.02)	<0.001
Hypoglycaemic agents	591 (12.02)	315 (13.94)	183 (12.96)	93 (7.47)	<0.001
All-cause mortality	1510 (30.71)	854 (37.79)	428 (30.31)	228 (18.31)	<0.001
Cardiovascular mortality	296 (7.99)	175 (11.07)	78 (7.34)	43 (4.06)	<0.001
Cerebrovascular mortality	83 (2.38)	51 (3.50)	22 (2.19)	10 (0.97)	<0.001

*LDL-C, low-density lipoprotein cholesterol; eGFR, estimated glomerular filtration rate. Antihypertensive drugs included angiotensin-converting enzyme inhibitors, angiotensin-II receptor blockers, and calcium channel blockers; Hypoglycaemic agents included oral drugs and insulin injection.*

*^a^Values for categorical and continuous variables are expressed as n (%) and median ± SD, respectively.*

### Association of Physical Activity and Risks of All-Cause and Cardiocerebrovascular Mortality

**FIGURE 2:**
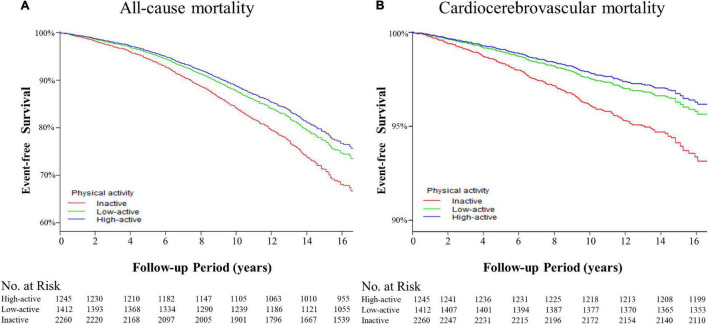
The Kaplan-Meier curves for event-free survival of the adverse outcomes among patients with hypertension. **(A)** The event-free survival rate for the all-cause mortality. High-active group vs. low-active group, *p* = 0.611; high-active group vs. inactive group, *p* < 0.001; low-active group vs. inactive group, *p* < 0.001 by log-rank tests. **(B)** Event-free survival rate for cardiocerebrovascular mortality. High-active group vs. low-active group, *p* = 0.975; high-active group vs. inactive group, *p* < 0.001; low-active group vs. inactive group, *p* < 0.001 by log-rank tests.

Kaplan-Meier survival curves were diverged according to MV-PA groups. The highest event-free survivals were observed in the high-active group in terms of all-cause and cardiocerebrovascular mortality (Figure 2) when compared with low-active (*p* > 0.05 for all outcomes) and inactive groups (*p* < 0.001 for all outcomes).

The multivariate Cox regression results are shown in Table 2. In the unadjusted Cox model, both the high-active and low-active groups had lower risks of all-cause [hazard ratio (HR): 0.44, 95% CI: 0.38–0.51, *p* < 0.001; 0.78, 95% CI: 0.70–0.88, *p* < 0.001; respectively] and cardiocerebrovascular mortality (0.35, 0.26–0.47, *p* < 0.001; 0.66, 0.52–0.84, *p* < 0.001; respectively) than the inactive group. After adjusting for age, gender, and race or fully adjusting for all covariates, the effects of high and low levels of MV-PA in reducing the risks of all-cause (0.70, 0.60–0.82, *p* < 0.001; 0.76, 0.68–0.86, *p* < 0.001; respectively) and cardiocerebrovascular mortality (0.56, 0.41–0.77, *p* < 0.001; 0.63, 0.50–0.81, *p* < 0.001; respectively) were significant. Moreover, similar results were observed in cases of independent studies of cardiovascular and cerebrovascular mortality in three Cox models.

**TABLE 2:** PA and risks for all-cause mortality and cardiocerebrovascular in patients with hypertension.

	Physical activity, MET-h/week				
Characteristics	Inactive (0)	Low-active (0 < to < 7.5)	*P*-value	High-active (≥7.5)	*P*-value
Participants, *n* (%)	2260 (45.96)	1412 (28.72)		1245 (25.32)	
**All-cause mortality**					
Event, n (%)	854 (37.79)	428 (30.31)		228 (18.31)	
Unadjusted HR (95% CI)	References	0.78 (0.70, 0.88)	<0.001	0.44 (0.38, 0.51)	<0.001
Age, sex, and race-adjusted HR (95% CI)	References	0.73 (0.65, 0.82)	<0.001	0.59 (0.51, 0.68)	<0.001
Fully adjusted HR (95% CI)^a^	References	0.76 (0.68, 0.86)	<0.001	0.70 (0.60, 0.82)	<0.001
**Cardiovascular mortality**					
Event, *n* (%)	175 (11.07)	78 (7.34)		43 (4.06)	
Unadjusted HR (95% CI)	References	0.66 (0.51, 0.87)	0.003	0.36 (0.26, 0.51)	<0.001
Age, sex, and race-adjusted HR (95% CI)	References	0.62 (0.47, 0.81)	<0.001	0.49 (0.35, 0.68)	<0.001
Fully adjusted HR (95% CI)^a^	References	0.64 (0.48, 0.84)	0.002	0.57 (0.40, 0.82)	0.003
**Cerebrovascular mortality**					
Event, *n* (%)	51 (3.50)	22 (2.19)		10 (0.97)	
Unadjusted HR (95% CI)	References	0.63 (0.38, 1.04)	0.071	0.28 (0.14, 0.55)	<0.001
Age, sex and race-adjusted HR (95% CI)	References	0.56 (0.34, 0.93)	0.025	0.40 (0.20, 0.79)	0.008
Fully adjusted HR (95% CI)^a^	References	0.51 (0.30, 0.88)	0.014	0.41 (0.20, 0.85)	0.017
**Cardiocerebrovascular mortality**					
Event, *n* (%)	226 (13.85)	100 (9.23)		53 (4.95)	
Unadjusted HR (95% CI)	References	0.66 (0.52, 0.84)	<0.001	0.35 (0.26, 0.47)	<0.001
Age, sex, and race-adjusted HR (95% CI)	References	0.62 (0.49, 0.79)	<0.001	0.48 (0.36, 0.66)	<0.001
Fully adjusted HR (95% CI)^a^	References	0.63 (0.50, 0.81)	<0.001	0.56 (0.41, 0.77)	<0.001

*MET, metabolic equivalent of task; h, hour; CI, confidence interval; HR, hazard ratio.*

*^a^Multivariable-adjusted models were adjusted for age, sex, body mass index, race, education, smoking, diabetes, coronary heart disease, chronic heart failure, stroke, cancer, systolic blood pressure, diastolic blood pressure, heart rate, total cholesterol, triglyceride, low-density lipoprotein cholesterol, eGFR, cholesterol intake, total fat intake, energy intake, protein intake, sodium intake, and medications.*

### Subgroup and Sensitivity Analyses of the Risks of All-Cause and Cardiocerebrovascular Mortality

**FIGURE 3:**
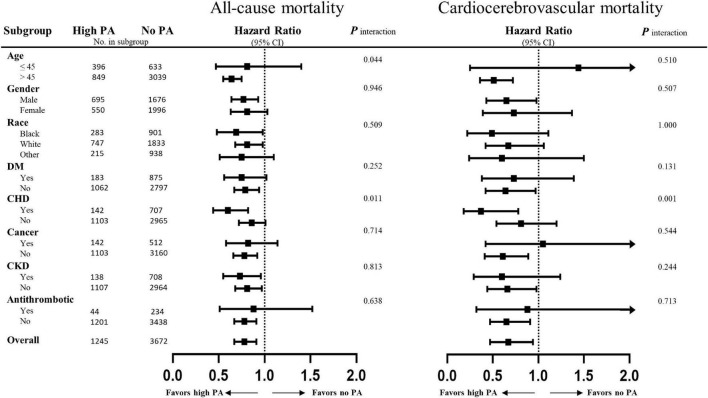
Subgroup analyses of the association between physical activity and the adverse outcomes. Risks of all-cause and cardiocerebrovascular mortality of the high physical activity defined by equals to or greater than 7.5 MET-h/week as compared to physically inactive cohorts. Results are expressed as multivariable-adjusted hazard ratio after controlling covariates that include age, gender, race, body mass index, education, smoking, diabetes, coronary heart disease, chronic heart failure, stroke, cancer, systolic blood pressure, diastolic blood pressure, heart rate, total cholesterol, triglyceride, low-density lipoprotein cholesterol, estimated glomerular filtration rate (eGFR), cholesterol intake, total fat intake, energy intake, protein intake, sodium intake, and medications (antiplatelet, warfarin, statin, antihypertensive drugs, β-blockers, α-blockers, diuretics, and hypoglycaemic agents), where possible interactions between above factors are also adjusted if necessary.

**FIGURE 4:**
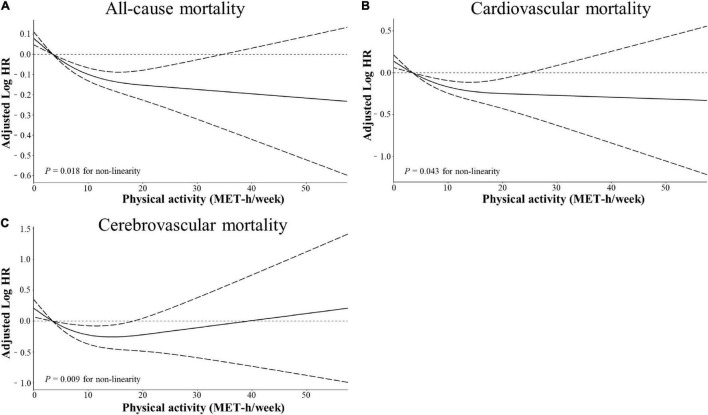
Restricted cubic spline models for the association between physical activity and adverse outcomes. Adjusted log_10_ hazards ratio (solid lines) and 95% confidence intervals (dashed lines) after controlling covariates in Table 1 for **(A)** all-cause mortality, **(B)** cardiovascular mortality, and **(C)** cerebrovascular mortality, compared with total physical activity volume quantified in metabolic equivalent hours per week (MET-h/week). Splines were examined in the fully adjusted model with the best placed knots at the 5, 35, 65, and 95th percentiles of physical activity level.

Stratified multivariate regression analysis was performed for the subgroups of study patients. Compared with the inactive group, the reduced risks of all-cause and cardiocerebrovascular mortality in the high-active and low-active groups were generally significant across all subgroups (Supplementary Table 1). The effect of MV-PA on lowering the risks of all study outcomes was modified only by age and coronary heart disease (Figure 3). In the sensitivity analysis, the results of propensity score matching supported the credibility of our findings that higher MV-PA might reduce the risks of study outcomes (Supplementary Tables 2, 3 and Figure 1). In addition, we further classified the patients into four groups based on MV-PA volume (0, 0 < to < 7.5, 7.5 to < 15, and ≥ 15 MET-h/week). The inverse association between higher MV-PA volume and lower risks of all-cause and cardiovascular mortality remained robust. However, the benefits did not increase in the highest quantile (≥ 15 MET-h/week) on the risk reduction of cerebrovascular mortality (Table 3). Furthermore, restricted cubic spline curves also confirmed that there were inverse relationships of MV-PA on all-cause and cardiovascular mortality and a U-shaped association for cerebrovascular mortality (Figures 4A–C).

**TABLE 3:** PA and risks for all-cause and cardiocerebrovascular mortality in four quantiles.

	Physical activity, MET-h/week			
Characteristics	0	0 < to < 7.5	7.5 to <15	≥15
Participants, *n* (%)	2260 (45.96)	1412 (28.72)	742 (15.09)	503 (10.23)
**All-cause mortality**				
Event, *n* (%)	854 (37.79)	428 (30.31)	159 (21.43)	14 (3.12)
Not adjusted HR (95% CI)	References	0.78 (0.70, 0.88)	0.53 (0.44, 0.62)	0.32 (0.25, 0.41)
Age, sex, and race-adjusted HR (95% CI)	References	0.73 (0.65, 0.82)	0.65 (0.55, 0.77)	0.49 (0.38, 0.62)
Fully adjusted HR (95% CI)^a^	References	0.76 (0.68, 0.86)	0.76 (0.63, 0.90)	0.59 (0.46, 0.76)
**Cardiovascular mortality**				
Event, n (%)	175 (11.07)	78 (7.34)	29 (4.74)	14 (3.12)
Not adjusted HR (95% CI)	References	0.66 (0.51, 0.87)	0.42 (0.29, 0.63)	0.28 (0.16, 0.48)
Age, sex, and race-adjusted HR (95% CI)	References	0.62 (0.47, 0.81)	0.52 (0.35, 0.77)	0.43 (0.25, 0.74)
Fully adjusted HR (95% CI)^a^	References	0.64 (0.48, 0.84)	0.60 (0.40, 0.91)	0.53 (0.30, 0.93)
**Cerebrovascular mortality**				
Event, *n* (%)	51 (3.50)	22 (2.19)	5 (0.85)	5 (1.14)
Not adjusted HR (95% CI)	References	0.63 (0.38, 1.04)	0.25 (0.10, 0.62)	0.33 (0.13, 0.83)
Age, sex, and race-adjusted HR (95% CI)	References	0.56 (0.34, 0.93)	0.32 (0.13, 0.80)	0.53 (0.21, 1.34)
Fully adjusted HR (95% CI)^a^	References	0.51 (0.30, 0.88)	0.36 (0.14, 0.93)	0.49 (0.18, 1.29)

*MET, metabolic equivalent of task; h, hour; CI, confidence interval; HR, hazard ratio. ^a^Multivariable-adjusted models were adjusted for age, sex, body mass index, race, education, smoking, diabetes, coronary heart disease, chronic heart failure, stroke, cancer, systolic blood pressure, diastolic blood pressure, heart rate, total cholesterol, triglyceride, low-density lipoprotein cholesterol, eGFR, cholesterol intake, total fat intake, energy intake, protein intake, sodium intake, and medications.*

## Discussion

The main findings in the present longitudinal cohort study are as follows: (1) generally, MV-PA was associated with protective roles in not only all-cause and cardiovascular mortality with inverse dose-response relationships but also cerebrovascular mortality with a U-shaped dose-response relationship; (2) a higher MV-PA of 7.5–15 MET-h/week was related to the lowest risks of cardiocerebrovascular and all-cause mortality. After the full adjustment of potential confounding factors such as age, gender, race, body mass index, education, smoking, comorbidities, kidney function, blood pressure, heart rate, lipid profiles, nutritional intake, and medications, such benefits of higher MV-PA remained significant. Although the effect of MV-PA interacted with age and coronary heart disease, the benefits of MV-PA were generally consistent across different subgroups of the cohort and remained robust in the sensitivity analysis. Previously, for patients with hypertension, most studies always focused on the effect of MV-PA to reduce blood pressure (13, 14). Recently, certain evidence suggested that MV-PA was associated with a better prognosis, regardless of the hypertension status (8). However, the safety and risks of patients with uncontrolled hypertension taking moderate and/or vigorous exercises have not yet been determined thoroughly. Therefore, the 2020 ESC Guidelines on Sports Cardiology and Exercise in Patients with Cardiovascular Disease indicate that a knowledge gap still exists on the safety and efficacy of MV-PA in hypertensive individuals (9). Overall, our findings could not only extend the current knowledge in this field but also further reveal a suitable dose range for the effectiveness of MV-PA in patients with hypertension.

One of our major findings, consistent with a previous study analysed by Park et al. (8) was that MV-PA was associated with reduced risks of cardiovascular and all-cause mortality in patients with hypertension. Compared to the Park et al’s study, we further adjusted diet style to include cholesterol, total fat, energy, protein, and sodium intake, and the results indicated that higher MV-PA might offset the adverse effect of a larger consumption of nutrition. Additionally, Park et al. found that, in the UK Biobank cohort, patients with controlled hypertension that were not engaged in any MV-PA particularly confronted with the highest risks of adverse outcomes, such as cardiac and all-cause death. In the general population, mounting evidence supports that longevity and cardiovascular benefits are related to MV-PA (15–17). All of the above evidence did show the positive benefits of MV-PA in both the general and hypertensive populations. Nonetheless, detailed dose-response relationships have not been determined in hypertensive populations. Moreover, cerebrovascular accidents and subsequent deaths are also prevalent in hypertension, so attention to prevent such harmful events may be necessary. To date, an investigation on the relationship between MV-PA and cerebrovascular death in a hypertensive population has not been conducted. In our study, higher MV-PA was related to the reduced cerebrovascular death in a U-shaped dose relationship. Most likely, PA could lower ischaemic and haemorrhagic stroke found in the studies by Park et al. and Feng et al. (18), and better-controlled blood pressure was observed in the present study, which eventually contributed to the reduced cerebrovascular mortality. However, we could not obtain detailed causes of cerebrovascular death from NHANES and evaluate the specified effect of MV-PA on different types of cerebrovascular death (i.e., haemorrhagic or ischaemic). It is worth noting that there seems to be a trend that hypertensive patients with old age over 45, diabetes, coronary heart disease, and chronic kidney disease might be more beneficial from higher MV-PA in the subgroup analyses. Traditionally, the age of 45 was considered a watershed threshold of increased cardiovascular risks (19). Indeed, in our study, the prevalence of comorbidities, such as diabetes, coronary heart disease, and chronic heart failure, in the > 45-year subgroup was 2.5–7 times that in the ≤ 45-year subgroup. In a previous report by Huerta et al. (20), for younger women and men of the general population, PA at work accounted for approximately 33 and 50% of total daily PA while household PA was approximately 75 and 13% of total non-occupational PA, so recreational PA might weakly contribute to reduce the risk of cardiovascular and overall mortality in the younger population. Hence, on the one hand, more recreational MV-PA should be encouraged to older patients with hypertension as their daily PA from work and household gradually declines with age (20, 21). On the other hand, sicker patients with these comorbidities are closely related to metabolic, inflammation, and endothelial dysfunction (4, 5) and they may therefore benefit greater from MV-PA. Clearly, the interaction between comorbidities and the detailed mechanism underlying the cardiocerebrovascular benefits of MV-PA in hypertension merit further investigation. Interestingly, patients in the present study with antiplatelet and/or anticoagulant treatment had over twofold more adverse outcomes than those without antithrombotic therapy. Subgroup and sensitivity analyses indicated that patients with hypertension without antithrombotic therapy might have more benefit from MV-PA. Previous studies revealed that regular PA was associated with increased platelet sensitivity to platelet inhibitors and weekly warfarin dose requirement in patients receiving antiplatelet and anticoagulant treatment, respectively, which meant an increased potential risk of bleeding (22, 23). Instead, one study disclosed that PA was beneficial for venous thromboembolism patients who received warfarin or heparin therapy (24). However, for hypertensive patients with antithrombosis, it is still unclear whether PA was associated with increased adverse outcomes. Further research on this topic is needed in the future.

Another finding was that high levels of MV-PA ≥ 7.5 or even ≥ 15 MET-h/week were associated with lower risks of all-cause and cardiovascular mortality. These inverse relationships were further confirmed by restricted cubic spline curves. Apparently, the near-infinitely beneficial relationship of higher MV-PA on all-cause and cardiovascular mortality should be cautious to confirm. Previous studies suggested that extreme intensity and volume of MV-PA might increase acute cardiovascular events, such as atrial and ventricular arrhythmia, myocardial fibrosis, and even sudden cardiovascular death (25–27). Conversely, high PA levels ≥ 7.5 MET-h/week were associated with a reduced risk of cerebrovascular death, and the benefits of MV-PA on cerebrovascular death appeared to reach a threshold of 15 MET-h/week. Notably, restricted cubic spline curves also confirmed this U-shaped association. Considering the paradoxical association between high MV-PA above 15 MET-h/week with increased risks of different study outcomes in our study, we proposed that an MV-PA volume of 7.5 to < 15 MET-h/week was associated with the lowest risks of cardiocerebrovascular and all-cause mortality in the hypertensive population.

The present study has some inevitable limitations. On the one hand, MV-PA volume was self-reported with a questionnaire, which might be subject to recall bias. Since accelerometer data were introduced and collected after the present study period, we failed to test the association between accelerometer results and the risks of study outcomes. In addition, although questionnaires are a common tool for epidemiological surveys and the instrument used in our study had been previously validated, we cannot deduce a causal relationship between MV-PA and mortality in hypertension. Comparatively, higher MV-PA as an independent favourable factor can be established. On the other hand, other sources of daily PA, such as work and households, were not included and the fluctuation of individual MV-PA volume was not considered during the follow-up duration, which might influence the quality of MV-PA information. Moreover, the additive effect of activity types on the risk reduction of outcomes in the same group was not explored. Finally, the causes of hypertension were unavailable from NHANES raw data, which indicated that a secondary form of hypertension might be included in the study. Some subgroups with a small number of events might exhibit a potentially statistical bias. Thus, the results should be interpreted with caution in clinical practise.

While acknowledging the inherent limitations, we insist that there are merits and clinical significance of our study. To our knowledge, the present study was the first to determine the optimal MV-PA volume with the lowest risks of cardiocerebrovascular and all-cause mortality in hypertension. Moreover, the MV-PA volume calculated by MET-h/week was convenient to translate into clinical practise. Second, we excluded some diseased individuals who were unable to engage in any PA to avoid possible reverse causation in the MV-PA assessment. More importantly, the large number of our cohort with exhaustive adjustment for potential confounders, sensitivity, and subgroup analyses was warranted to confirm our findings.

## Conclusion

In this large longitudinal cohort study with long-term follow-up, higher MV-PA was inversely associated with all-cause and cardiovascular mortality among patients with hypertension, which seems to support the “some is better than none, more is slightly better” approach. Additionally, a U-shaped association between MV-PA and cerebrovascular mortality could be observed. Further analysis confirmed that, for the hypertensive population, a minimum of 7.5 MET-h/week should be recommended while 15 MET-h/week appears to be the safe upper limit of MV-PA to avoid exercise-associated increases in the risk of cerebrovascular death. Overall, an optimal spectrum of MV-PA was 7.5 to < 15 MET-h/week to reduce the risks of all-cause and cardiocerebrovascular mortality in hypertension.

## Data Availability Statement

The raw data supporting the conclusions of this article will be made available by the authors, without undue reservation.

## Ethics Statement

The studies involving human participants were reviewed and approved by the US Centres for Disease Control and Prevention ratified the study protocols. The patients/participants provided their written informed consent to participate in this study.

## Author Contributions

J-PX, R-XZ, X-YM, SM, and M-ZZ conceived and designed the study. J-PX, Y-ZZ, and H-NL collected and analysed the data. J-PX drafted the manuscript. M-ZZ revised the manuscript. All authors have reviewed the final manuscript.

## Conflict of Interest

The authors declare that the research was conducted in the absence of any commercial or financial relationships that could be construed as a potential conflict of interest.

## Publisher’s Note

All claims expressed in this article are solely those of the authors and do not necessarily represent those of their affiliated organizations, or those of the publisher, the editors and the reviewers. Any product that may be evaluated in this article, or claim that may be made by its manufacturer, is not guaranteed or endorsed by the publisher.

## Footnotes

1
http://www.empowerstats.com
