# Supplementary material for: Analysis of the Dose-Response Effects of Physical Activity on Cardiocerebrovascular and All-Cause Mortality in Hypertension
Source: Front Cardiovasc Med. 2022 Mar 17;9:844680. doi: 10.3389/fcvm.2022.844680 (PMC8969098; doi:10.3389/fcvm.2022.844680)
Supplement: Supplementary file 1 [file Data_Sheet_1.docx]

| Table S1 Subgroup analysis between physical activity and risks for all-cause and cardiocerebrovascular mortality in patients with hypertension | | | | | | | |  |
| --- | --- | --- | --- | --- | --- | --- | --- | --- |
|  |  |  |  |  |  |  |  |  |
| Outcomes | No. of events | | | Low-active vs inactive | | High-active vs inactive | |  |
|  | Physical activity, MET-h/week | | |  |  |  |  |  |
|  | Inactive (0) | Low-active (0 < to < 7.5) | High-active  (≥ 7.5) | Unadjusted HR (95% CI) | Fully adjusted HR (95% CI) | Unadjusted HR (95% CI) | Fully adjusted HR (95% CI) |  |
| All-cause mortality | | | | | | | |  |
| Age ≤45 | 35 (8.79) | 12 (5.11) | 23 (5.81) | 0.57 (0.30, 1.10) | 0.88 (0.44, 1.76) | 0.69 (0.41, 1.17) | 0.78 (0.43, 1.40) |  |
| Age ＞45 | 819 (43.98) | 416 (35.34) | 205 (24.15) | 0.78 (0.69, 0.88) | 0.76 (0.67, 0.86) | 0.49 (0.42, 0.58) | 0.57 (0.49, 0.67) |  |
| Male | 431 (43.19) | 249 (36.73) | 148 (21.29) | 0.82 (0.70, 0.96) | 0.76 (0.64, 0.89) | 0.43 (0.36, 0.52) | 0.69 (0.56, 0.84) |  |
| Female | 423 (33.52) | 179 (24.39) | 80 (14.55) | 0.71 (0.60, 0.85) | 0.76 (0.64, 0.91) | 0.41 (0.32, 0.52) | 0.73 (0.56, 0.94) |  |
| Race, black | 215 (34.57) | 58 (20.79) | 39 (13.78) | 0.59 (0.44, 0.79) | 0.54 (0.40, 0.72) | 0.38 (0.27, 0.54) | 0.57 (0.39, 0.82) |  |
| Race, white | 441 (43.49) | 300 (36.63) | 156 (20.88) | 0.79 (0.69, 0.92) | 0.84 (0.72, 0.97) | 0.41 (0.34, 0.49) | 0.75 (0.61, 0.91) |  |
| Race, other | 198 (31.73) | 70 (22.29) | 33 (15.35) | 0.69 (0.53, 0.91) | 0.76 (0.57, 1.00) | 0.45 (0.31, 0.65) | 0.69 (0.46, 1.02) |  |
| Diabetes | 263 (47.99) | 132 (40.37) | 52 (28.42) | 0.81 (0.66, 1.00) | 0.73 (0.59, 0.91) | 0.53 (0.39, 0.71) | 0.66 (0.48, 0.91) |  |
| Without diabetes | 591 (34.52) | 296 (27.28) | 176 (16.57) | 0.77 (0.67, 0.89) | 0.77 (0.67, 0.89) | 0.44 (0.38, 0.53) | 0.71 (0.59, 0.85) |  |
| Coronary heart disease | 281 (65.35) | 142 (51.26) | 52 (36.62) | 0.70 (0.57, 0.85) | 0.72 (0.58, 0.88) | 0.43 (0.32, 0.58) | 0.51 (0.37, 0.71) |  |
| Without coronary heart disease | 573 (31.31) | 286 (25.20) | 176 (15.96) | 0.80 (0.69, 0.92) | 0.81 (0.70, 0.94) | 0.49 (0.41, 0.58) | 0.79 (0.66, 0.94) |  |
| Cancer | 167 (59.22) | 117 (50.87) | 49 (34.51) | 0.79 (0.63, 1.01) | 0.81 (0.62, 1.04) | 0.47 (0.34, 0.65) | 0.73 (0.51, 1.05) |  |
| Without cancer | 687 (34.73) | 311 (26.31) | 179 (16.23) | 0.74 (0.65, 0.84) | 0.74 (0.65, 0.85) | 0.43 (0.37, 0.51) | 0.69 (0.58, 0.83) |  |
| eGFR < 60 ml/min/1.73m2 | 287 (65.53) | 170 (62.96) | 65 (47.10) | 0.96 (0.79, 1.16) | 0.88 (0.72, 1.07) | 0.59 (0.45, 0.77) | 0.69 (0.51, 0.92) |  |
| eGFR ≥ 60 ml/min/1.73m2 | 567 (31.12) | 258 (22.59) | 163 (14.72) | 0.71 (0.61, 0.82) | 0.70 (0.60, 0.81) | 0.45 (0.38, 0.54) | 0.70 (0.58, 0.84) |  |
| Cardiocerebrovascular mortality | | | | | | | |  |
| Age ≤45 | 6 (1.63) | 1 (0.45) | 5 (1.32) | 0.28 (0.03, 2.29) | 0.23 (0.01, 4.49) | 0.84 (0.26, 2.76) | 0.95 (0.17, 5.27) |  |
| Age ＞45 | 220 (17.42) | 99 (11.51) | 48 (6.94) | 0.65 (0.52, 0.83) | 0.64 (0.50, 0.81) | 0.39 (0.28, 0.53) | 0.43 (0.31, 0.60) |  |
| Male | 127 (18.30) | 64 (12.98) | 38 (6.50) | 0.70 (0.52, 0.94) | 0.65 (0.48, 0.89) | 0.34 (0.23, 0.48) | 0.56 (0.38, 0.82) |  |
| Female | 99 (10.55) | 36 (6.09) | 15 (3.09) | 0.58 (0.40, 0.85) | 0.56 (0.38, 0.84) | 0.29 (0.17, 0.51) | 0.55 (0.30, 1.01) |  |
| Race, black | 57 (12.28) | 15 (6.36) | 9 (3.56) | 0.53 (0.30, 0.94) | 0.38 (0.20, 0.71) | 0.30 (0.15, 0.62) | 0.38 (0.18, 0.84) |  |
| Race, white | 123 (17.67) | 63 (10.82) | 35 (5.59) | 0.59 (0.44, 0.80) | 0.61 (0.44, 0.83) | 0.30 (0.20, 0.43) | 0.54 (0.36, 0.81) |  |
| Race, other | 46 (9.75) | 22 (8.27) | 9 (4.71) | 0.86 (0.52, 1.43) | 1.12 (0.63, 1.98) | 0.48 (0.24, 0.98) | 0.69 (0.31, 1.55) |  |
| Diabetes | 67 (19.03) | 42 (17.72) | 16 (10.88) | 0.94 (0.64, 1.38) | 0.80 (0.53, 1.19) | 0.56 (0.33, 0.97) | 0.63 (0.35, 1.13) |  |
| Without diabetes | 159 (12.42) | 58 (6.85) | 37 (4.01) | 0.54 (0.40, 0.73) | 0.54 (0.39, 0.74) | 0.32 (0.22, 0.45) | 0.53 (0.36, 0.79) |  |
| Coronary heart disease | 96 (39.18) | 44 (24.58) | 14 (13.46) | 0.59 (0.41, 0.84) | 0.60 (0.41, 0.88) | 0.29 (0.17, 0.51) | 0.29 (0.16, 0.55) |  |
| Without coronary heart disease | 130 (9.37) | 56 (6.19) | 39 (4.04) | 0.66 (0.48, 0.91) | 0.68 (0.49, 0.94) | 0.44 (0.30, 0.62) | 0.73 (0.50, 1.07) |  |
| Cancer | 41 (26.28) | 24 (17.52) | 12 (11.43) | 0.66 (0.40, 1.09) | 0.50 (0.26, 0.98) | 0.40 (0.21, 0.76) | 0.89 (0.40, 2.00) |  |
| Without cancer | 185 (12.53) | 76 (8.03) | 41 (4.25) | 0.63 (0.49, 0.83) | 0.64 (0.48, 0.84) | 0.33 (0.24, 0.47) | 0.51 (0.36, 0.74) |  |
| eGFR < 60 ml/min/1.73m2 | 82 (35.19) | 37 (27.01) | 16 (17.98) | 0.74 (0.50, 1.09) | 0.64 (0.41, 0.98) | 0.46 (0.27, 0.78) | 0.44 (0.24, 0.80) |  |
| eGFR ≥ 60 ml/min/1.73m2 | 144 (10.29) | 63 (6.65) | 37 (3.77) | 0.65 (0.48, 0.87) | 0.61 (0.45, 0.84) | 0.37 (0.26, 0.53) | 0.57 (0.38, 0.83) |  |
| MET: metabolic equivalent of task; h: hour; CI: confidence interval; HR: hazard ratio. | | | | | | | |  |

| Table S2 Baseline characteristics of study participants by physical activity after propensity score matching^a^ | | | | | | | | | |  |
| --- | --- | --- | --- | --- | --- | --- | --- | --- | --- | --- |
| Characteristics | Physical activity | | | | | | | | |  |
|  | Inactive (n=1412) | Low-active (n=1412) | *P*-value | Inactive (n=1084) | High-active (n=1084) | *P*-value | Low-active (n=1098) | High-active (n=1098) | *P*-value |  |
| Male | 612 (43.34) | 678 (48.02) | 0.013 | 545 (50.28) | 578 (53.32) | 0.156 | 555 (50.55%) | 597 (54.37%) | 0.073 |  |
| Age, years | 61.00 ± 15.55 | 61.50 ± 15.60 | 0.346 | 57.41 ± 16.19 | 55.34 ± 16.00 | 0.004 | 59.01 ± 15.82 | 55.41 ± 15.90 | <0.001 |  |
| Body mass index, kg/m2 | 30.66 ± 7.39 | 30.19 ± 6.42 | 0.418 | 30.28 ± 6.79 | 30.14 ± 6.38 | 0.933 | 30.13 ± 6.33 | 30.04 ± 6.37 | 0.605 |  |
| Race |  |  |  |  |  |  |  |  |  |  |
| Black | 407 (28.82) | 279 (19.76) | <0.001 | 286 (26.38) | 252 (23.25) | <0.001 | 239 (21.77%) | 240 (21.86%) | 0.377 |  |
| White | 653 (46.25) | 819 (58.00) |  | 539 (49.72) | 637 (58.76) |  | 639 (58.20%) | 663 (60.38%) |  |  |
| Other | 352 (24.93) | 314 (22.24) |  | 259 (23.89) | 195 (17.99) |  | 220 (20.04%) | 195 (17.76%) |  |  |
| Education |  |  |  |  |  |  |  |  |  |  |
| Lower than high school | 593 (42.00) | 422 (29.89) | <0.001 | 263 (24.26) | 200 (18.45) | 0.001 | 245 (22.31%) | 197 (17.94%) | 0.002 |  |
| High school | 371 (26.27) | 367 (25.99) |  | 277 (25.55) | 268 (24.72) |  | 290 (26.41%) | 257 (23.41%) |  |  |
| More than high school | 448 (31.73) | 623 (44.12) |  | 544 (50.18) | 616 (56.83) |  | 563 (51.28%) | 644 (58.65%) |  |  |
| Smoking |  |  |  |  |  |  |  |  |  |  |
| Never Smoker | 672 (47.59) | 688 (48.73) | <0.001 | 551 (50.83) | 510 (47.05) | <0.001 | 529 (48.18%) | 522 (47.54%) | 0.488 |  |
| Current Smoker | 438 (31.02) | 504 (35.69) |  | 306 (28.23) | 396 (36.53) |  | 383 (34.88%) | 406 (36.98%) |  |  |
| Ex-Smoker | 302 (21.39) | 220 (15.58) |  | 227 (20.94) | 178 (16.42) |  | 186 (16.94%) | 170 (15.48%) |  |  |
| Diabetes | 339 (24.01) | 327 (23.16) | 0.595 | 213 (19.65) | 178 (16.42) | 0.051 | 223 (20.31%) | 175 (15.94%) | 0.008 |  |
| Coronary heart disease | 264 (18.70) | 277 (19.62) | 0.534 | 164 (15.13) | 135 (12.45) | 0.071 | 179 (16.30%) | 137 (12.48%) | 0.011 |  |
| Chronic heart failure | 90 (6.37) | 99 (7.01) | 0.762 | 58 (5.35) | 34 (3.14) | 0.035 | 55 (5.01%) | 33 (3.01%) | 0.035 |  |
| Stroke | 97 (6.87) | 102 (7.22) | 0.844 | 66 (6.09) | 55 (5.07) | 0.078 | 65 (5.92%) | 53 (4.83%) | 0.316 |  |
| Cancer | 173 (12.25) | 230 (16.29) | 0.002 | 137 (12.64) | 129 (11.90) | 0.600 | 162 (14.75%) | 134 (12.20%) | 0.080 |  |
| Systolic blood pressure, mmHg | 130.94 ± 17.69 | 127.79 ± 13.46 | <0.001 | 128.99 ± 16.55 | 128.00 ± 14.03 | 0.267 | 127.44 ± 14.13 | 127.38 ± 13.87 | 0.828 |  |
| Diastolic blood pressure, mmHg | 74.24 ± 13.92 | 72.92 ± 12.28 | 0.010 | 74.35 ± 13.34 | 75.03 ± 12.19 | 0.116 | 73.93 ± 11.58 | 74.80 ± 11.88 | 0.010 |  |
| Heart rate, beats per minute | 73.07 ± 12.80 | 71.61 ± 12.29 | 0.008 | 72.41 ± 12.94 | 71.74 ± 13.49 | 0.208 | 71.89 ± 12.61 | 71.58 ± 13.71 | 0.400 |  |
| Total cholesterol, mg/dl | 206.90 ± 43.57 | 203.25 ± 42.48 | 0.020 | 203.76 ± 43.75 | 204.89 ± 43.27 | 0.439 | 203.86 ± 42.25 | 204.94 ± 43.64 | 0.654 |  |
| Triglyceride, mg/dl | 161.46 ± 72.51 | 162.29 ± 78.68 | 0.409 | 160.64 ± 76.48 | 159.55 ± 84.32 | 0.330 | 160.59 ± 79.57 | 159.87 ± 84.43 | 0.324 |  |
| LDL-C, mg/dl | 118.82 ± 25.86 | 118.95 ± 25.30 | 0.895 | 118.43 ± 24.85 | 118.91 ± 24.26 | 0.624 | 118.99 ± 25.01 | 119.16 ± 24.93 | 0.935 |  |
| eGFR, ml/min/1.73m^2^ | 84.22 ± 26.90 | 82.04 ± 24.84 | 0.013 | 85.72 ± 27.50 | 87.53 ± 23.24 | 0.149 | 84.05 ± 24.63 | 87.05 ± 23.02 | <0.001 |  |
| Cholesterol intake, mg | 274.24 ± 205.66 | 267.64 ± 193.05 | 0.423 | 283.17 ± 200.57 | 288.20 ± 221.56 | 0.989 | 276.33 ± 200.23 | 290.76 ± 223.47 | 0.155 |  |
| Total fat intake, gm | 69.84 ± 38.92 | 71.82 ± 39.24 | 0.226 | 74.98 ± 39.03 | 77.24 ± 40.65 | 0.291 | 74.28 ± 40.64 | 78.58 ± 42.32 | 0.006 |  |
| Energy intake, kcal | 1873.73 ± 866.14 | 1892.51 ± 825.07 | 0.257 | 1997.87 ± 839.80 | 2046.40 ± 859.48 | 0.136 | 1953.91 ± 857.87 | 2074.84 ± 883.70 | <0.001 |  |
| Protein intake, gm | 71.58 ± 36.34 | 74.32 ± 33.80 | 0.004 | 77.63 ± 36.99 | 80.66 ± 36.36 | 0.012 | 76.91 ± 35.14 | 82.03 ± 37.46 | <0.001 |  |
| Sodium intake, mg | 2941.07 ± 1184.91 | 3023.50 ± 1133.09 | 0.015 | 3044.04 ± 1182.93 | 3107.06 ± 1086.83 | 0.010 | 3040.36 ± 1152.59 | 3105.67 ± 1110.96 | 0.025 |  |
| Antiplatelet | 49 (3.47) | 55 (3.90) | 0.549 | 29 (2.68) | 28 (2.58) | 0.893 | 34 (3.10%) | 30 (2.73%) | 0.612 |  |
| Warfarin | 36 (2.55) | 37 (2.62%) | 0.906 | 23 (2.12) | 14 (1.29) | 0.136 | 24 (2.19%) | 14 (1.28%) | 0.102 |  |
| Statin | 214 (15.16) | 301 (21.32) | <0.001 | 183 (16.88) | 196 (18.08) | 0.462 | 219 (19.95%) | 210 (19.13%) | 0.628 |  |
| Antihypertensive drugs | 483 (34.21) | 499 (35.34) | 0.549 | 347 (32.01) | 332 (30.63) | 0.487 | 360 (32.79%) | 342 (31.15%) | 0.410 |  |
| β-blockers | 104 (7.37) | 135 (9.56) | 0.036 | 83 (7.66) | 86 (7.93) | 0.810 | 98 (8.93%) | 86 (7.83%) | 0.355 |  |
| α-blockers | 43 (3.05) | 48 (3.40) | 0.594 | 36 (3.32) | 28 (2.58) | 0.310 | 31 (2.82%) | 32 (2.91%) | 0.898 |  |
| Diuretics | 283 (20.04) | 298 (21.10) | 0.485 | 210 (19.37) | 175 (16.14) | 0.049 | 207 (18.85%) | 176 (16.03%) | 0.081 |  |
| Hypoglycemic agents | 201 (14.24) | 183 (12.96) | 0.323 | 101 (9.32) | 90 (8.30) | 0.405 | 115 (10.47%) | 91 (8.29%) | 0.079 |  |
| All-cause mortality | 524 (37.11) | 428 (30.31) | <0.001 | 333 (30.72) | 216 (19.93) | <0.001 | 283 (25.77%) | 223 (20.31%) | 0.002 |  |
| Cardiovascular mortality | 108 (10.84) | 78 (7.34) | 0.006 | 62 (7.63) | 41 (4.51) | 0.006 | 47 (5.45%) | 42 (4.58%) | 0.399 |  |
| Cerebrovascular mortality | 29 (3.16) | 22 (2.19) | 0.184 | 22 (2.85) | 10 (1.14) | 0.012 | 15 (1.81%) | 10 (1.13%) | 0.242 |  |
| LDL-C: low-density lipoprotein cholesterol. Antihypertensive drugs included angiotensin converting enzyme inhibitors, angiotensin-II receptor blockers and calcium channel blockers; Hypoglycemic agents included oral drugs and insulin injection.  ^a^Values for categorical and continuous variables are expressed as n (%) and median ± standard deviation, respectively. | | | | | | | | | |  |
|  |  |  |  |  |  |  |  |  |  |  |
|  |  |  |  |  |  |  |  |  |  |  |

| Table S3 Physical activity and risks for all-cause and cardiocerebrovascular mortality after propensity score matching | | | |  |
| --- | --- | --- | --- | --- |
|  |  |  |  |  |
| Outcomes | Physical activity, MET-h/week | | |  |
|  | Low-active vs inactive | High-active vs inactive | High-active vs low-active |  |
| All-cause mortality |  |  |  |  |
| Fully adjusted HR (95% CI)^a^ | 0.78 (0.68, 0.89) | 0.72 (0.60, 0.86) | 1.00 (0.83, 1.20) |  |
| Cardiovascular mortality |  |  |  |  |
| Fully adjusted HR (95% CI)^a^ | 0.68 (0.50, 0.93) | 0.62 (0.41, 0.95) | 1.13 (0.73, 1.76) |  |
| Cerebrovascular mortality |  |  |  |  |
| Fully adjusted HR (95% CI)^a^ | 0.63 (0.34, 1.17) | 0.32 (0.14, 0.73) | 0.98 (0.39, 2.48) |  |
| Cardiocerebrovascular mortality |  |  |  |  |
| Fully adjusted HR (95% CI)^a^ | 0.70 (0.53, 0.91) | 0.57 (0.40, 0.83) | 1.09 (0.74, 1.62) |  |
| MET: metabolic equivalent of task; h: hour; CI: confidence interval; HR: hazard ratio.  ^a^Multivariable-adjusted models were adjusted for age, sex, body mass index, race, education, smoking, diabetes, coronary heart disease, chronic heart failure, stroke, cancer, systolic blood pressure, diastolic blood pressure, heart rate, total cholesterol, triglyceride, low-density lipoprotein cholesterol, eGFR, cholesterol intake, total fat intake, energy intake, protein intake, sodium intake and medications | | | |  |
|  |  |  |  |  |
|  |  |  |  |  |
|  |  |  |  |  |
|  |  |  |  |  |


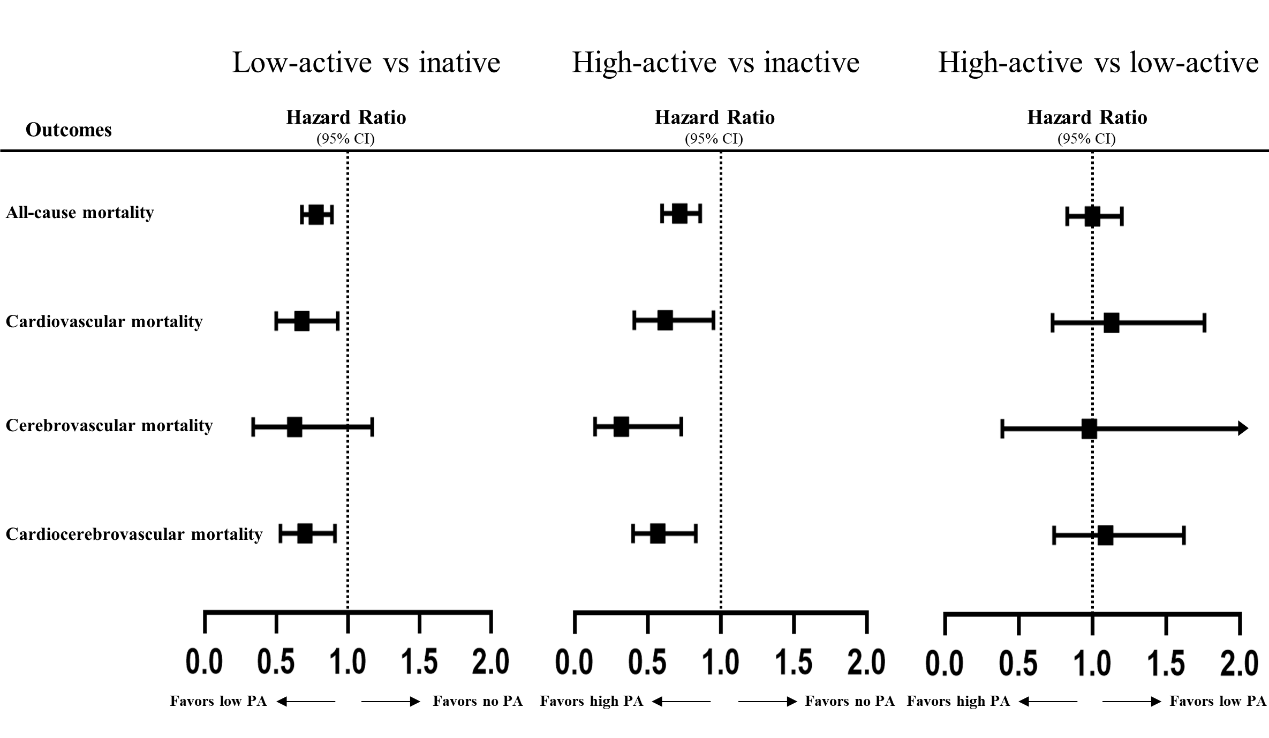


Figure S1 Physical activity and risks for all-cause and cardiocerebrovascular mortality after propensity score matching. PA: physical activity.
